# Research on the Mechanism of Asphalt Pavement Interlayer Bonding Reinforcement Based on Fiber Seal Coat

**DOI:** 10.3390/ma19142974

**Published:** 2026-07-10

**Authors:** Yu Wu, Yubi Zhao, Zhenhua Zhou, Hongzhi He

**Affiliations:** 1 Department of Road and Bridge Engineering, Sichuan Jiaotong Polytechnic University, Chengdu 611130, China; yuwu@svtcc.edu.cn; 2 School of Civil Engineering, Southwest Jiaotong University, Chengdu 610031, China; 3 School of Architecture Engineering and Planning, Jiujiang University, Jiujiang 332005, China; 2025012@jju.edu.cn; 4 School of Architecture Engineering, Sichuan University of Arts and Science, Dazhou 635000, China; 20240064@sasu.edu.cn

**Keywords:** asphalt pavement, fiber seal, parameter optimization, interlayer bonding, reinforcement mechanism

## Abstract

To investigate the reinforcement law and internal mechanism of fiber seals on the interlayer bonding performance of asphalt pavement, the SMA-13/AC-20 pavement structure was taken as the object to reveal the effects of fiber type (basalt fiber and glass fiber), dosage (0, 80, 100, and 120 g/m^2^), and length (3, 5, 7, and 9 cm). The control variable method and indoor direct shear and tensile tests were used to optimize the parameters. The results indicate that the optimal parameter combination is a fiber dosage of 100 g/m^2^ and a length of 5 cm. In this case, the reinforcement effect of basalt fiber is significantly superior to that of glass fiber, and the interlayer shear and tensile strengths reach 1.47 MPa and 0.793 MPa, respectively, which are 41.3% and 37.2% higher than those of the group without fiber. By forming a three-dimensional network structure, fibers exert a synergistic effect of reinforcement, bridging, and friction enhancement, constructing a robust interlayer composite system. This study provides experimental support for material optimization and multi-parameter collaborative design of a fiber seal coat.

## 1. Introduction

The interlayer bonding state of asphalt pavement is a key factor in determining its overall structural performance and service life. Weak or discontinuous interlayer interfaces are prone to inducing diseases such as slippage, delamination, and reflective cracks under environmental actions, accelerating the damage process of the pavement structure [1,2]. In conventional pavement construction, interlayer treatment mostly uses single-type bonding materials such as styrene–butadiene–styrene (SBS) or styrene–butadiene rubber (SBR)-modified asphalt [3,4], whose performance still has much room for improvement, and the treatment technology of composite seal coat is still insufficient. This calls for a deeper understanding of the performance of seal materials and their internal mechanisms.

In recent years, various fibers have been introduced into interlayer reinforcement. Basalt fiber and glass fiber are the most commonly used due to their high tensile strength and good thermal stability. These studies indicate that the optimal fiber dosage generally ranges from 80 to 120 g/m^2^ and the optimal length from 3 to 7 cm, varying with mixture type and test conditions [5,6,7,8]. However, most existing studies focused on a single fiber type or a single parameter, and systematic comparisons between basalt and glass fibers under identical conditions remain limited, especially for the SMA-13/AC-20 structure. This gap motivates the present study.

To date, no PRISMA-based systematic review has been published specifically on fiber-reinforced asphalt interlayers. This gap underscores the need for systematic parametric studies like the present work to provide fundamental data for future comprehensive reviews.

To develop more effective composite interlayer reinforcement techniques and thus achieve the long-life goal of pavement structures, many scholars have begun to study the performance optimization of interlayer materials. As a multifunctional layer composed of fibers, asphalt, and even chip stone [9,10,11], the fiber seal coat has attracted much attention for its active interlayer reinforcement technology. Numerous studies have shown that the introduction of fibers can significantly improve the high-temperature stability, low-temperature crack resistance, and fatigue resistance of asphalt mixtures [8,12,13].

Nevertheless, targeted, systematic research on fiber seal coats dedicated to boosting interlayer interfacial bonding is still inadequate. Existing literature seldom provides an in-depth dissection of the synergistic coupling effects of three key design variables (fiber type, dosage, and length), nor elaborates on their fundamental working mechanisms. Most existing studies on interlayer treatment focus on the characteristics of the reinforcing materials themselves [14,15], with little in-depth investigation of the reinforcement mechanism and the effects of factors. Current research on fiber-reinforced chip seal coat mainly focuses on the description of macroscopic phenomena, optimization of interlayer performance, and process improvement measures, and corresponding local technical standards have been issued [16,17]. However, the explanation of the underlying mechanism in these works is still not detailed enough. Moreover, most existing investigations adopt only one fiber type or fixed parameter combinations [18] and fail to thoroughly reveal the internal fiber reinforcement mechanism. Consequently, these findings cannot provide sufficient guidance for refined engineering design oriented toward performance optimization.

Based on this, this paper aims to improve the bonding performance of interlayer seals on asphalt pavements, selecting basalt fiber and glass fiber for comparative study. Through systematic parameter optimization tests, the optimal dosage and length of the two fibers are determined, and their reinforcement efficiency differences are compared under the optimal state. Combined with analysis of variance and interfacial mechanics theory, the reinforcement mechanism of fibers and the root cause of performance differences are revealed, aiming to establish a quantitative relationship of “fiber parameter—interlayer performance” and provide an experimental basis for material optimization and multi-parameter collaborative design of a fiber seal coat.

## 2. Experimental Program

### 2.1. Test Materials

In this study, SMA-13 was used for the upper layer of the composite specimen and AC-20 for the lower layer. The aggregate was basalt, and the mineral filler was limestone powder. Among these, SMA (Stone Mastic Asphalt) and AC (Asphalt Concrete) are standard asphalt mixture types according to JTG F40-2004 [19], where the numbers 13 and 20 denote the nominal maximum aggregate size in millimeters. The gradation compositions of the mixtures are shown in Table 1. SBS-modified asphalt was used as the binder in each mixture, and the asphalt used for interlayer bonding was also SBS-modified asphalt. Its key performance indices meet the requirements of the “Technical Specifications for Construction of Highway Asphalt Pavements” (JTG F40-2004) [19]. The specific performance indices are shown in Table 2. For interlayer seal coat treatment, basalt fiber (BF) and glass fiber (GF) were selected. Tests were conducted based on standards such as “Chopped Basalt Fiber for Cement Concrete and Mortar” (GB/T 23265-2023) [20] and “Glass Fiber Mats—Chopped Strand and Continuous Filament Mats” (GB/T 17470-2007) [21]. The basic performance indices of the fiber materials are shown in Table 3.

The materials used in this study include SBS-modified asphalt, basalt fiber, glass fiber, and aggregates. The SBS-modified asphalt was supplied by a road maintenance company in Sichuan Province. The basalt fiber and glass fiber were purchased from a new materials company. The aggregates used in the AC-20 and SMA-13 mixtures were sourced from a quarry in Dazhou, Sichuan. All materials meet the requirements of the relevant Chinese standards.

### 2.2. Specimen Preparation and Testing

To realistically simulate the actual compaction process of pavement and ensure the uniformity and repeatability of the fiber distribution state between layers, this study adopted a composite specimen preparation method of “rut plate forming and core drilling”. The specific steps are as follows:

(1) Forming the lower layer rut plate.

According to the target mix proportion of AC-20, the hot-mixed mixture (mixed at approximately 175–180 °C) was evenly loaded into a 300 mm × 300 mm × 50 mm rut mold and compacted using a wheel rolling machine at a compaction temperature of 160–165 °C. After compaction, the mold was cooled to room temperature and demolded to obtain an AC-20 lower-layer rut plate.

(2) Simulation of interlayer fiber seal coat construction.

A clean brush was used to remove dust from the surface of the AC-20 rut plate, and the plate was placed into a 300 mm × 300 mm × 100 mm rut mold. A precision balance and brush were utilized to spread SBS-modified asphalt evenly across the entire rutting plate surface following the designed spreading rate. Immediately afterwards, crushed stone and pre-weighed fibers cut to the target length were evenly spread on the asphalt surface along two perpendicular directions. The control group was spread without fibers. The spreading of SBS-modified asphalt between layers was precisely controlled as follows: first, the SBS-modified asphalt was heated in an oven using a small iron barrel; second, the rut plate specimen was placed on an electronic scale, and the surface was kept clean. Then, the electronic scale was zeroed, and the heated SBS-modified asphalt was evenly spread on the specimen surface with a brush and a wooden piece until the target weight was displayed on the electronic scale, which was the amount of SBS-modified asphalt applied.

(3) Forming the composite rut plate.

On the AC-20 rut plate with the fiber seal coat construction completed, the mixed SMA-13 mixture (mixed at approximately 175–180 °C) was evenly filled into the mold, and the same wheel rolling process at 160–165 °C as for the lower layer was applied for compaction, finally forming a composite rut plate structure of “SMA-13—fiber seal coat—AC-20”.

(4) Core drilling and processing of test specimens.

**Figure 1:**
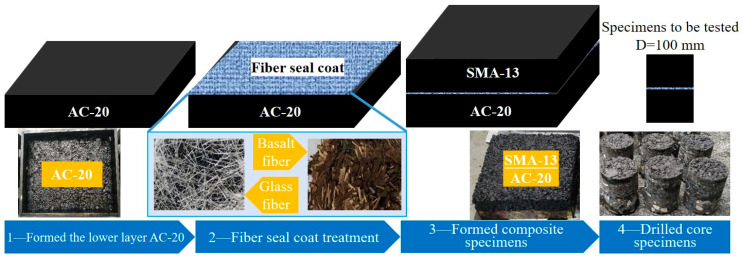
Preparation process of composite specimens.

After the composite rut plate was completely cooled, a water-cooled core drill was used to drill several cylindrical cores with a diameter of 100 mm at uniformly distributed positions along the plate plane. Finally, no fewer than three valid parallel specimens were prepared for each test condition (combination of fiber type × dosage × length). The entire specimen preparation process is shown in Figure 1.

**Figure 2:**
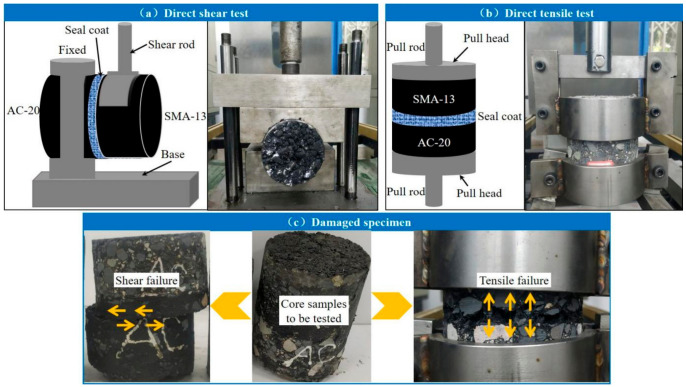
Schematic diagram of interlayer bonding strength test, loading devices, and failed specimens.

Based on the specimens prepared as above, the bonding strength was tested using a direct shear device and a direct tensile device. The schematic diagram of the tests, loading devices, and failed specimens is shown in Figure 2. During the test, the composite specimens to be tested were first placed in a temperature-controlled chamber to reach the corresponding temperature; then, the temperature-conditioned specimens were quickly installed on the test device; finally, the interlayer bonding strength test was completed using a universal testing machine. The loading rates of the shear and tensile tests were set at 50 mm/min and 10 mm/min, respectively [23].

### 2.3. Test Scheme

The tests were carried out in a laboratory environment at a constant temperature of 25 °C. Specimens were maintained in a thermostatically controlled oven at 25 °C for at least 4 h prior to testing to ensure a homogeneous internal temperature throughout the sample. This study set three variables: fiber type, fiber dosage, and fiber length. The interlayer working characteristics were explored based on indoor strength tests. The specific test scheme is shown in Table 4. A two-stage experimental design was adopted, and mechanism analysis was conducted based on the test results.

In the first stage, the control variable method was used. Based on existing research and preliminary test work, the fiber length was fixed at 5 cm, and the interlayer strength of basalt fiber under four dosages (0, 80, 100, and 120 g/m^2^) was investigated. Then, the optimal fiber dosage was fixed, and the interlayer strength under four lengths (3, 5, 7, and 9 cm) was investigated [13,23,24]. Through this stage, the theoretical optimal dosage and length of basalt fiber were determined. It should be noted that fibers were added to the interlayer as a reinforcement component rather than replacing any asphalt or aggregate constituent by mass or volume. The mixture proportions of the AC-20 lower layer and SMA-13 upper layer remained constant across all specimen groups.

In the second stage, based on the optimal parameter combination determined in the first stage, glass fiber was introduced as a reinforcing material, specimens were prepared and tested, and the reinforcement effects of the two fibers were compared.

## 3. Results and Analysis

### 3.1. Effect Analysis of Fiber Content on Interlayer Strength

**Figure 3:**
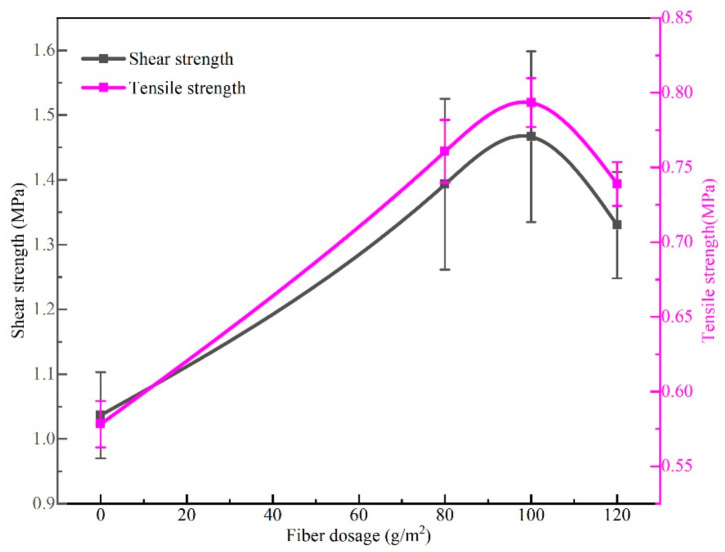
Effect of basalt fiber dosage on interlayer strength.

Regarding the effect of the fiber dosage on interlayer strength, to eliminate the influence of length, the fiber length was fixed at 5 cm, and the performance of basalt fiber under different dosages was evaluated using shear and tensile tests. The test results are shown in Figure 3.

Figure 3 indicates that basalt fiber exhibits a highly consistent enhancement effect on both interlayer shear strength and tensile strength; the curves of both strengths with fiber dosage present a clear unimodal shape, and the peak strength appears stably at 100 g/m^2^. When the dosage is below 100 g/m^2^, the strength increases rapidly with increasing dosage; when the dosage exceeds this value, the strength shows an obvious downward trend.

The essence of this law is the manifestation of the dynamic balance between the “network formation effect” and the “negative interface effect”. When the dosage is insufficient, the number of fibers is limited, making it difficult to form a continuous and effective three-dimensional reinforcement network; the reinforcement effect is weak, and the strength increase is slow. When the dosage reaches 100 g/m^2^, the fibers are evenly and reasonably distributed, overlapping each other to form a spatial network structure throughout the interface, which can efficiently transfer local stress to the entire area, restrain the relative slippage of aggregate and asphalt, and make the interlayer strength reach a peak. When the dosage exceeds 100 g/m^2^, the excess fibers break the dominance of the “network formation effect”, and the “negative interface effect” becomes prominent: on the one hand, the fibers are prone to agglomeration and entanglement, destroying the uniformity of distribution, and the agglomerated areas become weak spots and cause local stress concentration; on the other hand, the excess fibers have a large specific surface area and adsorb a large amount of asphalt mortar, resulting in a reduction in the thickness of the effective bonding film and deterioration of the bonding performance, ultimately reducing the interlayer strength.

This unimodal trend is consistent with findings reported by Lin et al. [8] and Rahman [24], who observed similar peak behavior for basalt and glass fibers, respectively. The consistency across different studies confirms that the trade-off between network formation and fiber agglomeration is a governing principle for fiber-reinforced interlayers.

### 3.2. Effect Analysis of Fiber Length on Interlayer Strength

**Figure 4:**
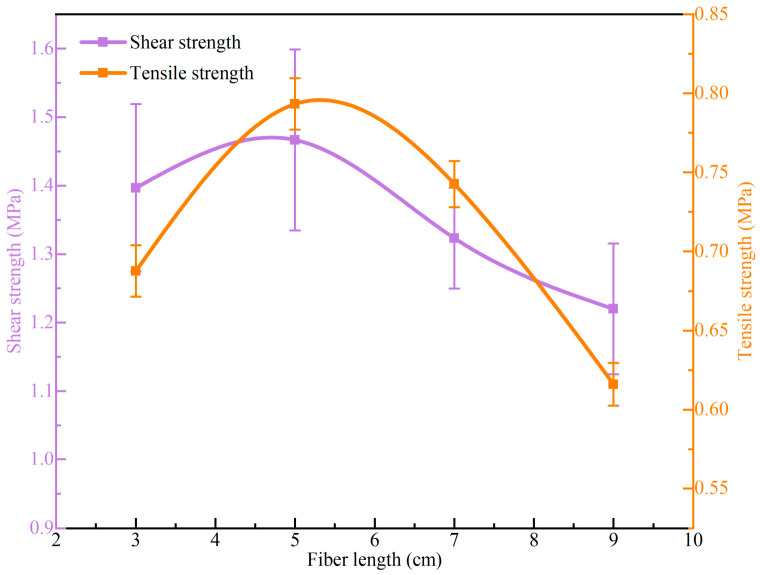
Effect of basalt fiber length on interlayer strength (with fixed dosage of 100 g/m^2^).

Under the premise of determining the optimal dosage of 100 g/m^2^ (as derived from the experiments in Section 3.1), the independent influence of the key geometric parameter of fiber length (3, 5, 7, and 9 cm) on interlayer performance was further investigated. The test results are shown in Figure 4.

As illustrated in Figure 4, the fiber length significantly affects the interlayer reinforcing effect. Interlayer strength increases first and then decreases with the growth of fiber length, and the optimum length is 5 cm. When the fiber is too short, the depth of embedment of both ends into the mixtures of the upper and lower layers is insufficient, and the anchorage mainly depends on interfacial chemical bonding, with weak mechanical interlocking; under load, it is easily pulled out entirely, and the high tensile strength cannot be utilized. When the length increases to 5 cm, the fibers can penetrate deeply into the aggregate gaps to obtain reliable mechanical interlocking while avoiding the problems of bending, tangling, and clumping that are prone to occur with overly long fibers, ensuring uniform distribution and effective formation of the three-dimensional network, achieving the best balance between “anchoring reliability” and “construction workability”. When the length further increases to 7 cm or even 9 cm, construction dispersion deteriorates sharply, easily forming local clumps and destroying interlayer uniformity; in addition, excessively long fibers are prone to micro-buckling instability under stress rather than ideal axial tension, and the actual load-bearing efficiency decreases.

This optimal length of 5 cm is consistent with the findings of Rahman et al. [24] for basalt fiber in RAC-13/RAC-20 interfaces, but differs from the 7 cm optimum reported by Gu [25] for basalt fiber in a different mixture. The underlying reason may be that the aggregate gradation and nominal maximum aggregate size determine the anchorage space available to the fibers.

### 3.3. Comparative Analysis of Fiber Types Based on Optimal Parameters

**Figure 5:**
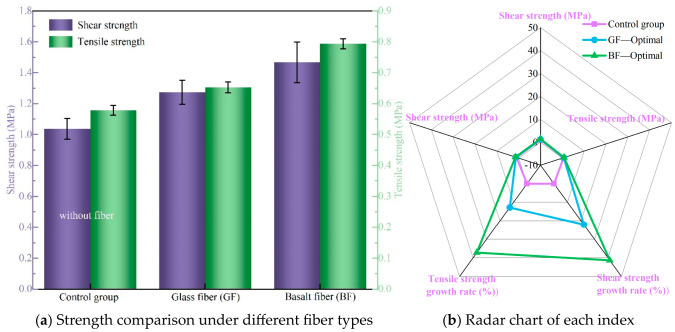
Comparison of reinforcement effects of fiber types on interlayer performance under optimal parameters.

After obtaining the optimal parameter combination of fibers, with a fiber dosage of 100 g/m^2^ and fiber length of 5 cm, the reinforcement effect differences in the two fiber materials themselves were compared and evaluated. The test results are shown in Figure 5.

It is evident from Figure 5a that inserting fibers into the interlayer remarkably enhances interlayer shear and tensile resistance. In all aspects, the interlayer reinforcement provided by basalt fibers outperforms glass fibers to a significant extent. Specifically, the shear strength (1.47 MPa) and tensile strength (0.793 MPa) of the BF-optimal specimen are about 15.7% and 21.6% higher than those of the GF-optimal specimen, respectively. More importantly, compared with the control group without fiber, the performance increase brought by BF (41.3% for shear, 37.2% for tension) is much higher than that of GF (22.1% for shear, 12.8% for tension).

The radar chart in Figure 5b intuitively compares the interlayer performance of different fiber systems from a multi-dimensional perspective. As observed, the contour area of the BF-optimal group extends the widest across shear strength, tensile strength, and strength growth rate metrics. This region fully surrounds and is clearly separated from the GF-optimal contour area. In comparison, both fiber groups feature much larger contour ranges than the fiberless control group, which is confined to the central region. This indicates that fiber incorporation produces a systematic improvement in interlayer performance, and the reinforcement efficiency of basalt fiber is more advantageous, especially in terms of the strength growth rate.

It should be noted that the standard deviations shown in Figure 5a reflect the inherent variability typical of composite material testing involving fiber dispersion and manual specimen preparation, which is common in this type of interlayer reinforcement study. Despite this variability, the ANOVA results presented in Section 4.1 (Table 5) confirm that the observed improvements are statistically significant (*p* < 0.01) and not attributable to random experimental errors. The magnitude of strength increase far exceeds the experimental scatter range.

This difference stems from the intrinsic properties of the two fibers and their interfacial interactions with the asphalt matrix. The chemical composition of basalt fiber has better compatibility with petroleum asphalt, forming a stronger fiber–asphalt interface, while the interfacial bonding of glass fiber mainly relies on physical attachment, which is relatively weak [26,27]. In addition, basalt fiber has a higher elastic modulus, can bear a larger proportion of stress under the same strain, and effectively restrains the plastic deformation of the asphalt matrix; its excellent high-temperature stability also ensures that the performance does not degrade during construction and service conditions. Therefore, the multi-phase interfacial system of “fiber-asphalt-aggregate” constructed by basalt fiber has a higher peak strength and better long-term durability.

## 4. Analysis of Reinforcement Mechanism Between Fiber Seal Coat

### 4.1. Statistical Significance Analysis

To quantitatively evaluate the significance of the effects of fiber type, dosage, and length on interlayer strength, a three-factor analysis of variance was performed based on all test data, with the *p*-value taken as 0.05, i.e., a confidence level of 95%. The ANOVA results for interlayer shear and tensile strengths are shown in Table 5 and Table 6.

It can be seen from Table 5 and Table 6 that the fiber type, dosage, and length all have highly statistically significant effects on the interlayer shear and tensile strengths. Based on the comparison of F statistics across factors, fiber dosage ranks first, followed by fiber length and fiber type in controlling shear strength. Regarding tensile strength, fiber type and fiber length exhibit substantially more significant effects, as indicated by their notably larger F-values compared with fiber dosage. This difference indicates that shear strength depends more on the macroscopic network structure and frictional effect dominated by dosage and length; tensile strength is more directly controlled by the interfacial bonding performance, so the fiber type, which determines the nature of the interface, and the length, which determines the anchorage effect, play a dominant role. This analysis confirms the necessity of optimizing fiber parameters from a statistical perspective and clarifies the primary and secondary relationships of different factors in resisting shear and tensile failure.

### 4.2. Fiber-Reinforced Mechanism

Based on the experimental results and ANOVA above, this section interprets the reinforcement mechanism from three interrelated aspects, with reference to existing literature.

(1) The randomly dispersed fibers overlap within the thin interlayer region (a few millimeters thick), forming a continuous three-dimensional network structure. This network transforms the homogeneous asphalt binder layer into a fiber-reinforced composite, which provides multiple force transmission paths [28]. When local stress concentration occurs, the network diffuses it to a wider area through fiber bridging, inhibiting microcrack propagation [29]. The optimal dosage of 100 g/m^2^ ensures sufficient fiber density for network connectivity without excessive entanglement; below this dosage, the network is incomplete, and reinforcement is weak; above it, agglomeration creates weak zones and stress concentrations.

(2) Fibers of appropriate length (5 cm) can penetrate into the surface voids of both upper (SMA-13) and lower (AC-20) layers, providing mechanical anchoring. This interlocking effect directly contributes to shear resistance by restraining relative slippage between layers. When fibers are too short (3 cm), the embedment depth is insufficient, and fibers are easily pulled out without mobilizing their tensile strength; when too long (≥7 cm), they tend to bend and tangle, reducing effective anchorage [30].

(3) The reinforcement efficiency also relies on the interfacial bonding between fiber and asphalt. A higher elastic modulus of basalt fiber allows it to bear greater stress under equal strain, restraining asphalt matrix plastic deformation [31].

**Figure 6:**
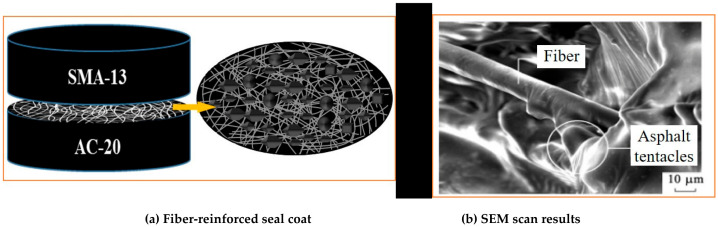
Combined effect and mechanism of fiber seal coat, Reference [32].

In summary, the optimal combination (basalt fiber, 100 g/m^2^, 5 cm) achieves the best balance among network connectivity, anchorage reliability, interfacial bonding, and constructability. As shown in Figure 6, the dispersed fibers overlap randomly within the interlayer to form a continuous three-dimensional network, transforming the homogeneous asphalt binder into a fiber-reinforced composite. According to SEM observations in Reference [32], fibers bridge across the asphalt phase, enhancing stress uniformity and overall stability. This network structure diffuses local stress concentrations over a larger area through bridging and creates multiple load-transfer pathways, effectively restraining aggregate-asphalt slippage and inhibiting microcrack propagation. Even in the event of localized failure, loads can be redistributed to prevent total interface failure. Therefore, the essence of optimizing fiber content and length is to construct an optimally connected, dense, reliably anchored, and uniformly distributed network within the limited interlayer space.

**Figure 7:**
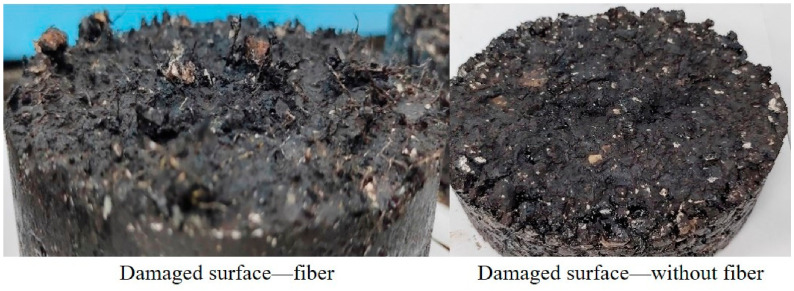
Comparison of interlayer failure interfaces with and without fibers.

On the other hand, the contribution of fibers to interlayer strength is multifaceted. For interlayer shear strength, the contribution comes mainly from three aspects: the direct shear resistance of the fibers themselves, the effective interlayer friction caused by the fibers, and the mechanical interlocking effect of fibers on the aggregates of the upper and lower layers. For tensile strength, the contribution mainly comes from the overall tensile capacity of the fiber network and the huge amount of energy consumed when the fibers are pulled out of the aggregate, which tends to rely more on the material properties of the fiber type itself. The comparison of shear failure interfaces in Figure 7 intuitively confirms the above mechanism. The failure interfaces of fiber-reinforced specimens are characterized by chaotic fracture morphology, which evidences the comprehensive reinforcing, anchoring, and crack-resisting mechanisms provided by the interwoven fiber network; while the failure interface of the specimen without fiber is relatively flat and clean, indicating that its failure occurs mainly in the interface itself, lacking the reinforcement of fibers.

Therefore, the key to the optimal design of a fiber seal coat lies in selecting a fiber type with excellent interfacial performance and high modulus, optimizing its spatial distribution parameters, and actively constructing a multi-scale, mechanically superior fiber-reinforced composite seal coat in the critical weak area between pavement layers, thereby comprehensively improving the strength, toughness, and durability of the interlayer bonding system.

### 4.3. Discussion on Engineering Application Value

This study reveals the optimal parameter combination and reinforcement mechanism of a fiber seal coat through indoor tests, which has certain guiding significance for practical engineering. From the perspective of a reinforcement effect, when basalt fiber is used at a dosage of 100 g/m^2^ and a length of 5 cm, the interlayer shear strength and tensile strength can be increased by 41.3% and 37.2%, respectively, which is significantly better than the 22.1% and 12.8% achieved by glass fiber. This finding aligns with the observation of Rahman et al. [24], showing that fibers can significantly improve interlayer strength, and that there is an optimal amount and length. During construction, the uniformity of fiber spreading should be strictly controlled to avoid local weak interfaces caused by fiber agglomeration or uneven distribution; it is recommended to use dedicated fiber spreading equipment and ensure that the asphalt spreading amount matches the fiber dosage to form a stable three-dimensional network structure.

In terms of economy, the unit price of basalt fiber is usually higher than that of glass fiber, but the resulting performance improvement is more significant, which can effectively extend the service life of the pavement and reduce later maintenance costs. For heavy-load traffic or long-life pavement projects, choosing basalt fiber may have better life-cycle economic benefits; while in projects with limited funding or lighter traffic loads, a glass fiber seal coat can also be used as an alternative for performance improvement. It should be noted that the economic analysis of actual projects needs to be comprehensively evaluated based on local material prices, construction conditions, and long-term performance observation data. The indoor results of this study provide basic support for subsequent field verification and economic comparisons.

## 5. Conclusions

On the basis of comprehensive experimental tests and statistical evaluation, the present research elaborates on the variation rules and coupled reinforcement mechanisms of three key parameters (fiber type, dosage, and length) affecting interlayer bonding performance in asphalt pavement structures. The main conclusions are as follows:

(1) The optimal parameter combination of fibers is a dosage of 100 g/m^2^ and a length of 5 cm. In this optimal parametric state, interlayer shear and tensile strengths attain their peak magnitudes, realizing a favorable balance between the formation of a three-dimensional fiber reinforcement network and practical constructability.

(2) The reinforcement effect of basalt fiber is better than that of glass fiber. Under the optimal parameters, its shear and tensile strengths are 15.7% and 21.6% higher, respectively, compared with the control group without fiber; the increase brought by basalt fiber (41.3% for shear, 37.2% for tension) is significantly higher than that of glass fiber (22.1%, 12.8%).

(3) Analysis of variance shows that the fiber dosage, length, and type all have highly significant effects on interlayer strength (*p* < 0.01). Shear strength is dominated by dosage and length, while tensile strength is more sensitive to type and length. This difference reflects that shear resistance relies primarily on a macroscopic network structure and friction, whereas tensile resistance depends more on interfacial bonding quality and anchorage effectiveness.

(4) The reinforcement mechanism involves three synergistic effects: three-dimensional network formation, fiber anchorage in aggregate voids for mechanical interlocking, and fiber–asphalt interfacial adhesion. The optimal combination (basalt fiber, 100 g/m^2^, 5 cm) achieves the best balance among these effects, transforming the homogeneous asphalt bonding layer into a fiber-reinforced composite with improved interlayer integrity and load-transfer efficiency.

(5) It should be pointed out that the conclusions of this paper are based on indoor standard curing conditions and, as such, lack field verification. When extended to practical engineering, targeted field tests are still needed. This study is limited to static normal-temperature conditions and does not consider dynamic loads or environmental coupling effects. Further work is expected to clarify the performance evolution characteristics under multi-field coupled environments, elucidate the microscale reinforcing mechanism, and conduct economic evaluations, thereby advancing the durable application of fiber seal coat reinforcement technology.

## Figures and Tables

**Table 1:** Gradation statistics of surface layer mixtures.

Mixture Type	Percentage Passing Through Each Sieve (mm)/%											
	26.5	19	16	13.2	9.5	4.75	2.36	1.18	0.6	0.3	0.15	0.075
SMA-13	100	100	100	95	62.5	27	20.5	19	16	13	12	10
AC-20	100	95	85	71	61	41	30	22.5	16	11	8.5	5

**Table 2:** Basic performance indices of SBS-modified asphalt [22].

Test Index	Test Result	Reference Value	Test Method (JTG E20)
Penetration, 25 °C, 5 S, 100 g (0.1 mm)	55	40–60	T0604
Ductility, 5 °C (cm)	26	≥20	T0605
Softening point (°C)	68	≥60	T0606
Kinematic viscosity 135 °C (Pa·s)	1.79	≤3	T0620
Elastic recovery 25 °C (%)	83	≥75	T0662

**Table 3:** Basic performance indices of fiber materials.

Performance Index	Density (g/cm^3^)	Tensile Strength (MPa)	Elastic Modulus (GPa)	Elongation (%)	Diameter (um)	Water Absorption (%)
Basalt fiber	2.75	4500	101	3.2	17	1.5
Glass fiber	2.55	3400	71	3.37	15	1.3

**Note:** Density is defined as mass per unit volume of the solid fiber material.

**Table 4:** Test scheme setup.

Parameter	Variable	Number of Levels
Influencing factors	Fiber type	Basalt fiber, Glass fiber
	Fiber dosage (g/m^2^)	0, 80, 100, 120
	Fiber length (cm)	3, 5, 7, 9

**Table 5:** ANOVA results for interlayer shear strength.

Influencing Factor	SS	DF	MS	*F* Value	*p* Value
Fiber type	0.08	1	0.08	10.239	0.003
Fiber dosage	0.48	3	0.16	20.351	0.000
Fiber length	0.405	4	0.101	12.9	0.000
Error	0.267	34	0.008		
Total	10.379	43			

**Table 6:** ANOVA results for interlayer tensile strength.

Influencing Factor	SS	DF	MS	*F* Value	*p*-Value
Fiber type	0.037	1	0.037	150.722	0.000
Fiber dosage	0.093	3	0.031	125.014	0.000
Fiber length	0.144	4	0.036	145.411	0.000
Error	0.008	34	0.000		
Total	2.838	43			

## Data Availability

The original contributions presented in this study are included in the article. Further inquiries can be directed to the corresponding author.
